# Pandemie- und Seuchengeschichte als Pflegegeschichte?

**DOI:** 10.1007/s00048-020-00252-w

**Published:** 2020-05-12

**Authors:** Karen Nolte

**Affiliations:** Institut für Geschichte und Ethik der Medizin, Ruprecht-Karls-Universität Heidelberg, Im Neuenheimer Feld 327, 69120 Heidelberg, Deutschland

**Keywords:** COVID-19, Pandemien, Pflegegeschichte, Krankenpflege, Historiographie, COVID-19, Pandemics, Nursing History, Nursing, Historiography

## Abstract

Dieser Beitrag ist Teil des Forums COVID-19: Perspektiven in den Geistes- und Sozialwissenschaften. In der aktuellen COVID-19-Pandemie wird die Bedeutung von professioneller Krankenpflege weithin anerkannt. In der deutschsprachigen und internationalen Forschung ist die Geschichte der Krankenpflege bei Pandemien und Epidemien weitgehend ungeschrieben. Der vorliegende Beitrag gibt einen Überblick über Fragen und Ergebnisse in diesem Forschungsbereich und diskutiert das Potenzial einer pandemischen Krankenpflegegeschichte.

## Abstract (en)

This paper is part of Forum COVID-19: Perspectives in the Humanities and Social Sciences. In the current COVID 19 pandemic, the importance of professional nursing is widely recognized. In German-speaking and international research, the history of nursing during pandemics and epidemics is largely unwritten. This paper gives an overview of questions and results in this research area and discusses the potential of a pandemic nursing history.

In der Öffentlichkeit und Politik wird derzeit die fundamentale gesellschaftliche Bedeutung von Pflege weithin anerkannt. Denn für die Genesung von COVID-19-Infizierten mit schweren Verläufen, deren Erkrankung medizinisch nicht ursächlich behandelt werden kann, ist der Beitrag professioneller Pflege – besonders bei zu beatmenden Patient*innen – zentral. Doch auch in der Sorge für Menschen der sogenannten „Risikogruppe“ – alte und Menschen mit Vorerkrankungen – in der ambulanten und stationären Pflege zu Hause und in Altenpflegeeinrichtungen sind Pflegefachkräfte besonders gefordert. Zugleich scheint der zuvor berufspolitisch angegangene Kampf des Deutschen Berufsverbands für Pflegeberufe (DBfK) für eine Verbesserung der Arbeitsbedingungen und höhere Bezahlung – besonders im Bereich der Altenpflege – nun mit Verweis auf die Krise von der Politik in eine Warteschleife gestellt zu sein. Pflegepersonaluntergrenzen wurden im Eilverfahren rückwirkend zum 1. März 2020 vorübergehend außer Kraft gesetzt und im Gegenzug administrative Tätigkeiten reduziert. Pflegekammern und der DBfK weisen auf die krisenhafte Zuspitzung zuvor schon prekärer Arbeitsbedingungen, mangelnde Schutzausrüstung und die Überlastung von Pflegefachkräften hin.

Bezeichnenderweise ist in der aktuellen Ausgabe von „Die Schwester/der Pfleger“ (Herzog 2020) ein Artikel abgedruckt, der mit „Stille Helden“ betitelt die aktuelle Situation von Pflegenden im Kampf gegen das Coronavirus international vergleichend thematisiert. Dass das offizielle Organ des DBfK den Topos des „Stillen Helden“ bemüht, gibt zu denken. Offenbar ist das aus dem 19. Jahrhundert stammende christliche Konzept der Selbstverleugnung (Nolte 2013) in der Pflege noch sehr präsent. Die Vorstellung von der „stillen“ Pflegearbeit hat wohl auch dazu beigetragen, dass die Geschichte der Pflege in den historischen Pandemien und Seuchen sowohl in der deutschsprachigen als auch internationalen Forschung eine weitgehend ungeschriebene ist: Pflege wird bis heute als quasi natürliches „weibliches Arbeitsvermögen“ (Ostner 1991) wahrgenommen. Wohl wegen der durch Geschlechterhierarchien geprägten gesellschaftlichen Abwertung von Pflege ist die Leistung von professionell Pflegenden bei der Bekämpfung von Seuchen und Pandemien bislang wenig erforscht.

Im ersten Schritt wird ein Überblick über Fragestellungen und Forschungsergebnisse gegeben, der sich auf Forschungen der deutschsprachigen Pflegegeschichte und der angelsächsisch geprägten *Nursing History* von Pandemien bzw. Seuchen im 19. und 20. Jahrhundert konzentrieren wird. Über den Pflegealltag in der Vormoderne wissen wir kaum etwas, dementsprechend ist die Bedeutung von Pflege während der Seuchen in dieser Zeit noch nicht untersucht und kann daher hier nicht diskutiert werden. Im zweiten Schritt werden die Potentiale dieses Forschungsgebiets aufgezeigt und diskutiert.

Die sogenannte Spanische Grippe von 1918–1920, die eigentlich in Kansas/USA ihren Ursprung hatte, wird in deutschsprachigen Überblickswerken zum Ersten Weltkrieg und seinen Folgen überwiegend nicht als historisches Ereignis angesehen, das ausführlich darzustellen sei (Rengeling 2017: 20). Diese Influenza-Pandemie, die somit nicht Teil des kollektiven Gedächtnisses geworden ist, stieß erst mit den Influenza-Epidemien des 21. Jahrhunderts und besonders in den letzten Wochen im Zeichen von COVID-19 auf viel öffentliches Interesse. Die durchaus übersichtliche deutschsprachige Forschungsliteratur zu dieser Pandemie spart den Beitrag von Pflegenden zur Bekämpfung dieser Pandemie und deren Arbeitsbedingungen weitgehend aus (Michels 2010; Witte 2008). Auch die in Deutschland insgesamt noch junge Pflegegeschichtsforschung (Nolte 2012) hat sich bislang kaum mit der Rolle von Pflegenden in historischen Pandemien des 20. Jahrhunderts befasst. In der noch überschaubaren Forschungsliteratur zur Krankenpflege im Ersten Weltkrieg, wird nur von Stölzle der Alltag der „Seuchenpflege“ untersucht: Hier geht es die immer wiederkehrenden Epidemien wie Typhus, Pocken, Cholera sowie endemisch die auftretenden Infektionskrankheiten wie Tuberkulose, Malaria sowie venerische Krankheiten in der Zeit vor der Influenza 1918–1920. Die dichte Beschreibung des täglichen Umgangs von Pflegenden mit einer Vielfalt von tödlichen Infektionskrankheiten gibt einen Eindruck von der Erfahrung, die vermutlich später deren Umgang mit Influenzakranken prägte. Stölzle arbeitet im Detail heraus, wie das noch junge bakteriologische Wissen auch unter den schwierigen Bedingungen des Lazaretts in der Etappe bereits dem pflegerischen Handeln implizit war (Stölzle 2013: 56–66). So ist aus Braunschweigs lokalhistorischen Forschungen zum Einsatz von Diakonissen während der Spanischen Grippe im Schweizer Basel zu ersehen, wie entscheidend die zuvor etablierte hygienische Ausbildung und eine gründliche Krankenbeobachtung in der Pflege von Grippekranken waren (Braunschweig 2019).

Pflegehistorische Forschungen zur sogenannten Spanischen Grippe, die ihren Ursprung eigentlich in Kansas hatte, wurden in der nordamerikanischen *Nursing History* jeweils durch drohende Pandemien – „Vogelgrippe“ (Robinson 1990) und „Schweinegrippe“ (Keeling 2010) – angeregt. Diese Publikationen heben vor allem die Bedeutung einer eigenen pflegerischen Professionalität im Umgang mit der Pandemie hervor. Während in Robinsons Studie die zentrale Bedeutung von *registered nurses*^1^ in der kommunalen Gesundheitsversorgung hervorgehoben wird, geht Keelings Untersuchung eher auf die Rolle der Pflegeorganisationen bei der Rekrutierung und Verteilung von Pflegepersonal zur Versorgung der an Spanischer Grippe Erkrankten ein. Beide Studien heben hervor, dass die *skills* professionell Pflegender für eine erfolgreiche Behandlung der an Grippe Erkrankten entscheidend waren. Dies entsprach auch durchaus dem Selbstbild der zu der Zeit bereits gut ausgebildeten *registered nurses*, die um 1900 zum Teil schon einen akademischen Abschluss hatten. Robinson charakterisiert in ihrer Studie den Einsatz von ausgebildeten Pflegenden während in den Jahren 1918 und 1919 der Pandemie als eine Geschichte der Professionalisierung: Neu gegründete lokale *Visiting Nurses Associations* versetzten Krankenschwestern in die Lage, eigenständig ambulante Pflegemaßnahmen nach damaligen professionellen Standards in Familien durchzuführen und als Expertinnen, die (noch) Gesunden in sanitärer und hygienischer Prävention von Infektionen zu unterweisen (Robinson 1990: 21–22). Wood hingegen zeigt für Neuseeland, dass die massenhafte Rekrutierung von Laienpflegerinnen während der Influenza-Pandemie in den Jahren 1918 und 1919 den Status der *registered nurses* bedrohte und somit vorübergehend eine Entwicklung der Deprofessionalisierung von Pflege in Gang setzte, gegen die sich das Pflegefachpersonal öffentlichkeitswirksam wehrte (Wood 2016).

In einer deutschsprachigen populärwissenschaftlichen Darstellung zur „Spanischen Grippe“ ist eine Vielzahl an Fotografien abgedruckt, auf denen Pflegende abgebildet sind. Dieses Material stammt fast ausschließlich aus den USA – diese Quellen werden weder analysiert, noch differenziert oder kontextualisiert. Pflegende werden somit nur auf der visuellen Ebene in die Geschichtsschreibung integriert (Salfellner 2018). Für eine noch zu leistende fotogeschichtliche Rekonstruktion der Pflegegeschichte während der Influenza-Pandemie 1918–1920 in den USA gäbe es mithin eine Fülle von Quellenmaterial.

Deutsche Forschungen konzentrieren sich – ebenfalls motiviert durch die Frage nach der Professionalisierung des Pflegeberufs – auf andere Pandemien, nämlich die Cholera-Pandemien in Deutschland im 19. Jahrhundert. Die Cholera-Pandemie von 1830/31 zog nicht nur die Gründung der Krankenpflegeschule an der Berliner Charité, sondern auch einen regelrechten „Kongregationsfrühling“ nach sich, das heißt viele Gründungen von katholischen Pflegeorden. Auch die erste 1836 Diakonissenanstalt in Kaiserswerth kann mit der „Cholera Asiatica“ in Zusammenhang gebracht werden, nachdem in den Lazaretten und Hospitälern ein Mangel an „verständigen“ Pflegekräften offensichtlich geworden war (Wolff 2002: 66–75; Büttner 2016).

Derzeit lenken New Yorker Pflegefachkräfte, die mit Müllsäcken und einfachem OP-Mundnasenschutz bekleidet vor die Presse treten, die Aufmerksamkeit darauf, dass sie COVID-19-Infizierte ungenügend geschützt pflegen müssen. Auch in Deutschland werden Atemschutzmasken und Schutzkleidung in Krankenhäusern derzeit mehrfach verwendet, in der Langzeitpflege fehlte Schutzkleidung von Beginn der Pandemie an. Anders als in anderen von COVID-19 betroffenen Ländern treten deutsche Pflegefachkräfte in den Medien kaum als Akteur*innen in Erscheinung, um auf ihre Gefährdung hinzuweisen. Die Gefahren, der Pflegende sich bei der Versorgung von Infektionskranken während historischer Pandemien aussetzten, und die Arbeitsbedingungen werden in der Pflegegeschichtsforschung kaum thematisiert. Hähner-Rombachs Forschung zu Ansteckung und Sterblichkeit von Krankenschwestern in der Pflege von Tuberkulosekranken nimmt diesen Aspekt der Geschichte von Infektionskrankheiten in den Blick. Sie weist auf die besondere Exposition von Krankenschwestern hin und betont die gravierenden sozialen Auswirkungen für Pflegende, wenn sie an Tuberkulose erkrankten: Als Berufsgruppe wurden sie erst 1929 in die bereits 1889 eingeführte Unfallversicherung aufgenommen, obwohl ihre Gefährdung zu erkranken von Experten schon um 1900 als sehr hoch eingeschätzt worden war (Hähner-Rombach 2009). Braunschweig kann zeigen, dass die Schwächung durch endemisch auftretende Tuberkuloseerkrankungen Krankenschwestern während der Influenza-Pandemie in den Jahren 1918 und 1919 zu einer Risikogruppe werden ließ, was sie jedoch nicht davon abhielt, ihre Pflicht in den Lazaretten zu erfüllen (Braunschweig 2019: 28). Anschließend an diese Publikationen könnte in der historischen Forschung zur Gesundheitsfürsorge herausgestellt werden, unter welchen Bedingungen gesundheitliche Risiken der Pflegenden problematisiert wurden. Pflegende waren und sind in ihrer Arbeit beständig gefährlichen Krankheitserregern ausgesetzt. Zentral für den Umgang mit dem Infektionsrisiko war und ist die intensive Schulung in Hygiene. Dieses praktische Wissen ist nicht nur für den Selbstschutz in der Pflege notwendig.

Die Erforschung der historischen Genese hygienischer Ausbildung von Pflegenden erscheint naheliegend mit Blick auf den Umstand, dass bei den Influenza-Pandemien Prävention in Form von Hygiene in den Fokus ihrer Bekämpfung rückte, wenn weder Impfstoffe noch wirksame Therapien verfügbar waren. In Anlehnung an Boses kulturwissenschaftliche Studie zum gegenwärtigen „Verhältnis von Sauberkeit, Macht und Arbeit im Krankenhaus“ (Bose 2017) ließe sich mit einer praxeologischen Fragestellung das *Doing* von Hygiene in der Krankenpflege historisch untersuchen. Neben Krankenpflegelehrbüchern, Pflegefachzeitschriften und Unterrichtsmanuskripten von Krankenpflegeschulen wäre Oral History der Generation, die in der zweite Hälfte des 20. Jahrhunderts in der Pflege arbeiteten, gewinnbringend.

Pflegehistorische Forschung zu den noch wenig untersuchten Pandemien des 20. und 21. Jahrhunderts wäre vielversprechend, um die komplexe gesellschaftliche und soziale Situation von Pflegenden in den Blick zu nehmen und aufzuzeigen, wie Krankenpflege gesellschaftlich wahrgenommen wurde, über welche Ressourcen Pflegende verfügten und welche Schwachstellen und Defizite sich in der extremen Situation der Pandemie offenbarten.

Bereits 1957 zur Zeit der „Asiatischen Grippe“ spitzte sich der Mangel an Pflegefachkräften in der BRD krisenhaft zu, da das alte Modell des Mutterhaussystems nicht mehr genügend Nachwuchs generierte (Kreutzer 2005). Während man in Westdeutschland die Pflegeausbildung als Reaktion auf den „Schwesternmangel“ nur langsam reformierte, wurde dieselbe in der DDR in den 1950er Jahren der Facharbeiterausbildung gleichgestellt und bereits in den 1970er Jahren akademisiert (Thiekötter 2006). Bis heute wurde das Problem des seit den 1950er Jahren bestehenden Fachkräftemangels in der Pflege nicht grundsätzlich angegangen, so dass sich derzeit unter den Bedingungen der COVID-19-Pandemie der weiterhin bestehende Pflegefachkräftemangel deutlich auswirkt. Im Hinblick auf die mediale Öffentlichkeit sowie fachliche und politische Überlegungen wäre zu rekonstruieren, wie der Stellenwert von Pflege während der Pandemien der 1950er und 1960er Jahre eingeschätzt und diskutiert wurde sowie welche gesundheitspolitischen Konsequenzen aus den Pandemien gezogen wurden. Unter der Perspektive einer Pandemiegeschichte der Pflege und ihrer Rolle in der öffentlichen Gesundheitsfürsorge wäre auch die Arbeit in der Gemeindepflege und in der damals noch existierenden Schulkrankenpflege zu untersuchen.

Abschließend lässt sich festhalten, dass jeweils aktuelle Bedrohungen durch Pandemien den Blick auf deren Geschichte und somit Forschung in dem Bereich motivierten. Mit dem Fokus auf eine gesamtgesellschaftliche Krisensituation kann die Untersuchung von Pflegegeschichte gewinnbringend für einen kritischen Blick auf die Geschichte des Gesundheitswesens sein und zeigen, worin jeweils der Kern professioneller Pflege gesehen wurde. Das heißt, durch die öffentliche Aufmerksamkeit entstehen in der Ausnahmesituation einer Pandemie möglicherweise Quellen, die Einblick in die Situation Pflegender und deren Arbeitsbedingungen und somit auch in den jeweiligen Zustand des Gesundheitswesens gewähren.

## Footnotes

1 Registered nurses hatten in den USA schon damals eine dreijährige Ausbildung, zum Teil sogar einen akademischen Abschluss einer *Nursing School* an einer Universität.

## References

[CR2] von Bose K (2017). Klinisch rein. Zum Verhältnis von Sauberkeit, Macht und Arbeit im Krankenhaus.

[CR3] Braunschweig S (2019). „Opfer treuer Pflichterfüllung“. Der Einsatz des Pflegepersonals bei der Grippe-Epidemie 1918 und 1919. Krankenpflege. Soins Infirmiers. Cure infermieristische.

[CR4] Büttner A, Vögele J, Knöll S, Noack T (2016). Konfessionelle Schwestern in der Cholerapflege. Epidemien und Pandemien in historischer Perspektive. Epidemics and Pandemics in Historical Perspective.

[CR5] Hähner-Rombach S, Hähner-Rombach S (2009). Kranke Schwestern. Umgang mit Tuberkulose unter dem Pflegepersonal 1890 bis 1930. Alltag in der Krankenpflege: Geschichte und Gegenwart. Everyday Nursing Life: Past and Present.

[CR6] Herzog U (2020). Stille Helden. Die Schwester/Der Pfleger. Die Fachzeitschrift für professionell Pflegende.

[CR8] Keeling AW (2010). “Alert to the Necessities of the Emergency”: U.S. Nursing During the 1918 Influenza Pandemic. Public Health Reports.

[CR9] Kreutzer S (2005). Vom „Liebesdienst“ zum modernen Frauenberuf. Die Reform der Krankenpflege nach 1945.

[CR10] Michels E (2010). Die Spanische Grippe 1918/19. Verlauf, Folgen und Deutungen in Deutschland im Kontext des Ersten Weltkriegs. Vierteljahrshefte für Zeitgeschichte.

[CR12] Nolte K (2012). Einführung: Pflegegeschichte – Fragestellungen und Perspektiven. Medizinhistorisches Journal.

[CR11] Nolte K, D’Antonio P, Fairman J, Whelan J (2013). Protestant Nursing Care in Germany in the 19th Century – Concepts and Social Practice. Handbook for the Global History of Nursing.

[CR13] Ostner I (1991). „Weibliches Arbeitsvermögen“ und soziale Differenzierung. Leviathan.

[CR14] Rengeling D (2017). Vom geduldigen Ausharren zur allumfassenden Prävention. Grippe-Viren im Spiegel von Wissenschaft, Politik und Öffentlichkeit.

[CR15] Robinson KR (1990). The Role of Nursing in the Influenza Epidemic of 1918–1919. Nursing Forum.

[CR16] Salfellner H (2018). Die Spanische Grippe. Eine Geschichte der Pandemie von 1918.

[CR17] Stölzle A (2013). Kriegskrankenpflege im Ersten Weltkrieg. Das Pflegepersonal der freiwilligen Krankenpflege in den Etappen des Deutschen Kaiserreiches.

[CR18] Thiekötter A (2006). Pflegeausbildung in der Deutschen Demokratischen Republik. Ein Beitrag zur Berufsgeschichte der Pflege.

[CR19] Witte W (2008). Tollkirschen und Quarantäne. Die Geschichte der Spanischen Grippe.

[CR20] Wolff H-P (2002). Studien zur deutschsprachigen Geschichte der Pflege.

[CR21] Wood PJ (2016). Managing Boundaries between Professional and Lay Nursing Following the Influenza Pandemic, 1918–1919: Insights for Professional Resilience Today. Journal of Clinical Nursing.

